# Regulatory mechanisms of microRNA expression

**DOI:** 10.1186/s12967-016-0893-x

**Published:** 2016-05-20

**Authors:** Lyudmila F. Gulyaeva, Nicolay E. Kushlinskiy

**Affiliations:** Research Institute of Molecular Biology and Biophysics, Timakov St., 2/12, Novosibirsk, 630117 Russia; Novosibirsk State University, Pirogova 2, Novosibirsk, 630090 Russia; The Russian Oncological Scientific Center of N. N. Blochin of Ministry of Health of the Russian Federation, Kashirskoye Highway 24, Moscow, 115478 Russia

**Keywords:** MicroRNA expression, Gene expression, Hypermethylation, Cancer, MiRNA processing, Cytokines, Xenobiotics, Nuclear receptors

## Abstract

MicroRNAs (miRs, miRNAs) are small molecules of 18–22 nucleotides that serve as important regulators of gene expression at the post-transcriptional level. One of the mechanisms through which miRNAs regulate gene expression involves the interaction of their “seed” sequences primarily with 3′-end and more rarely with 5′-end, of mRNA transcribed from target genes. Numerous studies over the past decade have been devoted to quantitative and qualitative assessment of miRNAs expression and have shown remarkable changes in miRNA expression profiles in various diseases. Thus, profiling of miRNA expression can be an important tool for diagnostics and treatment of disease. However, less attention has been paid towards understanding the underlying reasons for changes in miRNA expression, especially in cancer cells. The purpose of this review is to analyze and systematize current data that explains reasons for changes in the expression of miRNAs. The review will cover both transcriptional (changes in gene expression and promoter hypermethylation) and post-transcriptional (changes in miRNA processing) mechanisms of regulation of miRNA expression, as well as effects of endogenous (hormones, cytokines) and exogenous (xenobiotics) compounds on the miRNA expression. The review will summarize the complex multilevel regulation of miRNA expression, in relation to cell type, physiological state of the body and various external factors.

## Background

**Fig. 1:**
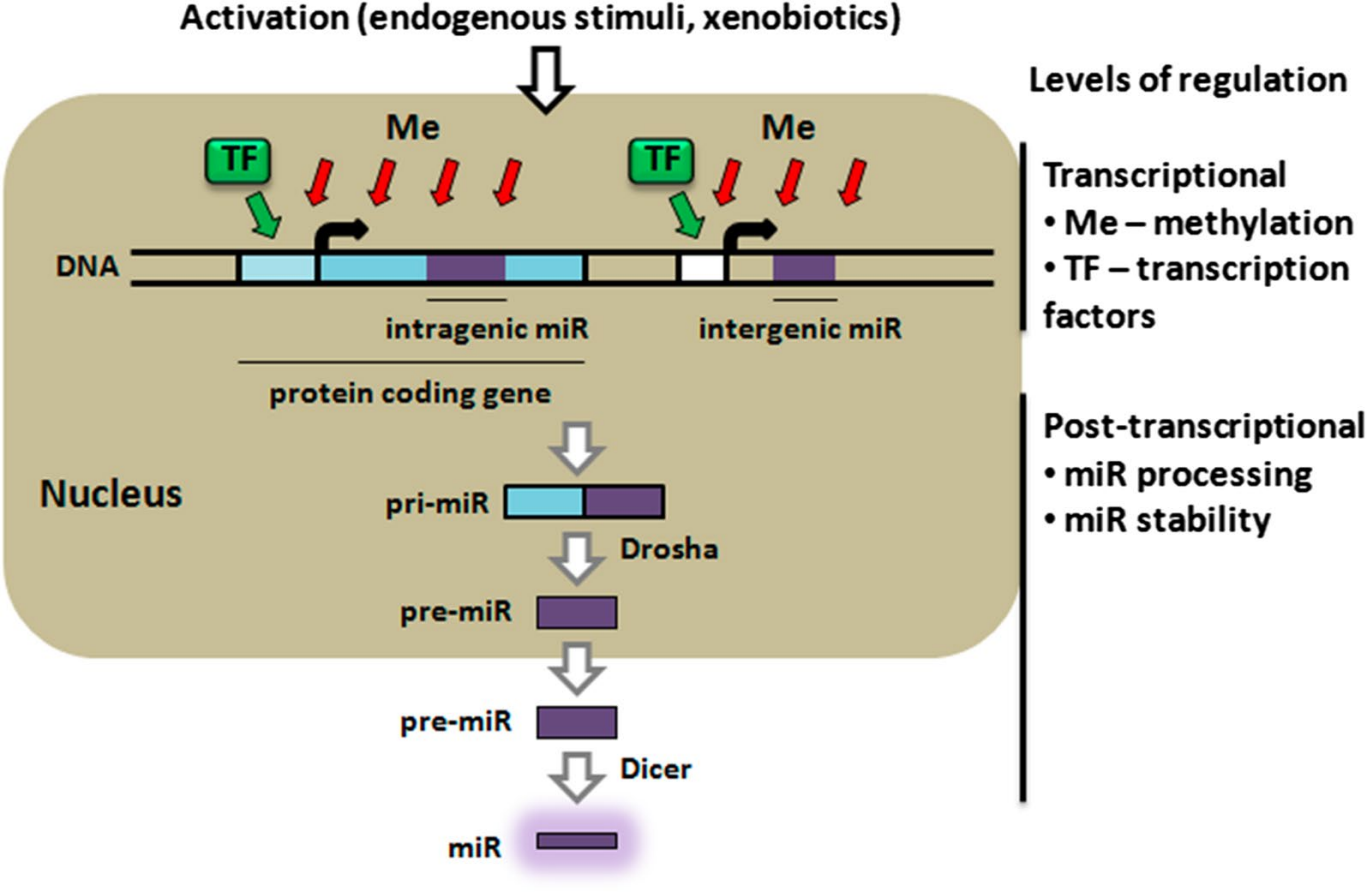
Levels of regulation of microRNA expression

MicroRNAs (miRs, miRNAs) represent a new class of single-stranded RNAs of 18–22 nucleotides, which play a key regulatory role in gene expression at the posttranscriptional level. One of the key mechanisms of their action is interaction of their “seed” sequences with 3′-end, and more rarely with 5′-end, of mRNA transcribed from target genes, followed by degradation of target mRNA. The reduction in the amount of a specific mRNA is an important result of this molecular event. MiRs are important regulatory molecules in many biological processes. Numerous studies over the past decade have been devoted to quantitative and qualitative assessment of miRNA expression and have shown considerable changes in their expression profiles in various diseases [1–4]. The most remarkable changes were observed in cancer [5–8]. The results of these studies point to the profiling of miRNA expression as an important tool for diagnostics and treatment of diseases. However, knowledge of the underlying reasons and mechanisms behind changes in miRNA expression is still limited. A clear understanding of the molecular mechanisms regulating miRNA expression would allow an explanation of the variations in the expression of protein coding genes. Here we suggest a generalized scheme of both transcriptional and post-transcriptional regulation of microRNA expression (Fig. 1). Transcriptional regulation includes changes in the expression of a host gene and hypermethylation of the promoter of host or microRNA genes. Post-transcriptional mechanisms represent changes in miRNA processing and stability. In addition, the effects of endogenous (hormones, cytokines) and exogenous (xenobiotics) compounds on the expression of miRNAs are considered. This review summarizes the complex multilevel regulation of miRNA expression in relation to cell type, physiological state of the body and various external factors.

## Defects in the miRNA processing

Biogenesis or processing of miRNAs has been extensively studied by many groups in recent years [9, 10]. MiRNAs can be transcribed by RNA Polymerase II together with the host gene (intragenic miRNAs) or independently of the host gene with the use of their own promoter (intergenic miRNAs). MiRNA’s primary transcripts or pri-miRNAs have a unique structure that differs from the rest of RNA, which is represented by a hairpin and three spiral turns, flanked by the segments of a single stranded RNA. This can be recognized by a so-called microprocessor complex that contains RNA- binding protein DGCR8 and RNase III Drosha. This complex cuts pri-miRNAs and generates a pre-miRNA of 60 nucleotides, representing the structure of the truncated hairpin with a two- nucleotide ledge and a two- spiral midstream. This allows miRNA to interact with Exportin-5 and Ran GTPase in order to be further sequestered into the cytoplasm. In the cytoplasm this protein-RNA complex is recognized by RNase III Dicer that cuts pre-miRNA into a mature form of miRNA. Interestingly, it has been shown that Drosha generates a smaller variety of miRNAs than Dicer [9] and, therefore, the ultimate content of miRNAs is dependent upon the activity of these enzymes. The factors regulating the expression of microRNAs at the stage of processing remain under investigation. For example, Pawlicki and Steitz [11] observed that pri-miRNA transcripts retained at transcription sites due to the deletion of 3′-end processing signals are converted into precursor miRNAs (pre-miRNAs) more efficiently than pri-miRNAs that are cleaved, polyadenylated, and released.

For some exonic miRNAs, such as miR-155, miR-22 and miR-146a, both unspliced and spliced transcripts may serve as primary miRNA transcripts. They are partly localized in the cytoplasm and thus not fully available for processing to the mature miRNAs [12]. The splicing and transport to the cytoplasm may represent a novel mechanism to regulate cellular exonic miRNA levels and function.

Changes in miRNA expression profiles could also be caused by changes in pre-miRNA export into the cytoplasm, impaired activity of Dicer/TRBP complex or post-translational changes in the Ago family of proteins [13, 14].

Hata and Kashima [5] have reviewed and analyzed various defects in miRNA biogenesis and some examples of changes in the activity of microprocessor Drosha. A variety of somatic and germ line mutations in the genes encoding enzymes involved in miRNA biogenesis, such as Drosha, DGCR8, Xpo5, Dicer1 and TRBP, were identified in malignant tumors [13, 15]. Somatic missense mutations affecting the RNase IIIb domain of *DICER1* in nonepithelial ovarian cancers were shown [16]. The authors concluded that this is a novel mechanism through which the perturbation of microRNA processing may be oncogenic.

The authors emphasize the importance of studying the components of biosynthetic pathways in miRNA biogenesis, in order to identify novel regulatory molecules that could serve as potential targets for therapeutic intervention. Another important contributor to miRNA levels in the cell is the stability of miRNA, which depends on the stage of development or cell type involved. Towler and colleagues [17] have reviewed several mechanisms controlling the stability of miRNA. One example is the simple but effective mechanism, whereby a few nucleotides (often adenosines) are added to the 3′-end of miRNA, thus promoting its stability. Wyman and co-authors [18] indentified multiple enzymes, including MTPAP, PAPD4, PAPD5, ZCCHC6, ZCCHC11, and TUT1, which participate in this 3′ nucleotide non-templated addition to miRNAs. The modifications result predominantly in adenylation and uridylation and are seen across tissue types, disease states, and developmental stages.

Another mechanism is the presence of sequences such as AU/UA within particular miRNAs that destabilize their secondary structure. The percentage of AU or UA dinucleotide (in either the 5′–3′ or 3′–5′ orientation), but not the total percentage of A + U, strongly correlated with the half-life of select miRNAs abundant in brain [19].

A few proteins have been discovered that can bind miRNAs and prolong their half-life. It has also been suggested that interaction of miRNA with its target mRNA or with ncRNAs can also affect stability of miRNAs. It was shown that HuR, a member of the ElaV family of RNA-binding proteins, may suppress the inhibitory effect of miRNAs [20]. Argonaute proteins play an important role in the regulation of microRNA expression and function. It was shown that Argonautes elevated mature miRNA expression post-transcriptionally, independently of RNase activity [21]. In addition, overexpression of Argonaute proteins decelerated miRNA degradation and increased miRNA half-life [22].

Finally, miRNA stability can be regulated by specific ribonucleases. Recently, Segalla and colleagues [23] has shown that ribonuclease DIS3 may regulate the levels of the tumor suppressor let-7 miRNAs.

Editing of miRNA may also alter miRNA processing following the changes in Ago complex and target mRNA binding. Adenosine to inosine (A-to-I) RNA editing of miR catalyzed by adenosine deaminase acting on RNA (ADAR) proteins may affect the stability, biogenesis, and target recognition of microRNA leading to changes in gene expression [24]. Yang and co-authors [25] has shown that mature miRNA-142 expression levels increased substantially in ADAR1 null or ADAR2 null mice. The authors discussed a new function of RNA editing in the control of miRNA biogenesis.

Adenosine deaminases can cause the changes in miRNA abundance and sequence during embryogenesis, as demonstrated in transgenic mouse embryos [26]. Using high-throughput RNA sequencing, Ekdahl and co-authors have shown the increased editing in mature miRNA from the mouse transcriptome during brain maturation [27]. There is no doubt today that microRNA editing plays a significant role in the regulation of miRNA activity. Kawahara and colleagues [28] estimated that approximately 16 % of human pri-miRNAs are subjects to A-to-I editing. Other enzymes like DEAD-box RNA helicase 6, DDX6 (p54/RCK) can contribute to post-transcriptional down-regulation of miR-143/145 expression by prompting the degradation of its host gene product, NCR143/145 RNA in cancer cells [29].

All of the mechanisms above have been proposed based on studies in animal models or human cell lines; the relevance to the human disease state, therefore, remains a subject for future investigations.

## Co-expression of miRNAs with host genes

Changes in intragenic miRNA expression can occur due to changes in the expression of host genes where the miRNA is encoded. Several studies have suggested links between miRNA expression and transcription factors, host genes and targets of mRNAs in various malignant tumors [7, 30]. However, experimental proof of this correlation is still lacking. Li and co-authors [31] analyzed miRNA expression, transcription factors and their gene targets in non-small cell lung cancer. Authors utilized several databases of TF-miRNA feed forward loops (FFLs) and chose FFLs typical of cancer and showed miR-17 (associated with transcription factors E2F1 and RB1) as an important family in the regulatory network of interaction between miRNA and transcription factors. Another study demonstrated dependence of miRNA expression on a host gene [32]. The authors summarized experimental data as well as observations showing changes in miRNA expression in response to various stimuli from the immune system. Two particular miRNA expressed in B cells were found- miR-155-5p and miR-155-3p. These miRNAs are located within the Integration Cluster gene (BIC) and are induced in response to various stimuli from the immune system. This emphasizes the importance of this type of study because of the role of miE-155-3p in the immune response and development of malignant tumors. Another study showed co-expression of miR-208a and its host gene *myh6* in rats with heart failure exposed to a 7-day treatment with isoproterenol [33]. Another interesting aspect is co-expression of miRNAs with other miRNAs. Recent studies utilizing transcriptome analysis, including that of Chaulk and coworkers [34], showed that miRNAs are mostly clustered and expressed as a single pri-miRNA transcript. For example, miR-11 and miR-998 have recently been shown to be organized in a cluster and have a co-dependent expression pattern [35].

Indeed, both intra- and intergenic miRNAs can be regulated by numerous transcription factors. For instance, transcription factors HNF1β and HNF1α differently regulate serum microRNAs in patients with diabetes [36]. Using a genome-wide RNA sequencing analysis, Mitxelena and colleagues [37] identified E2F7- regulated miRNAs and a new regulatory network that involves transcriptional and post-transcriptional mechanisms controlled by E2F7 and miRNAs.

Using target miRNA and TF-miRNA interactions from different databases, many investigators create regulatory networks for various diseases. Differentially expressed genes and miRNAs play key roles in this network. So, renal cell carcinoma was classed according to the regulatory association of adjacent nodes in the three network levels for comparing and analyzing the interacting features of each differentially expressed gene. Two of these genes, PTEN and TP53, showed a particular feature of regulating miRNAs and being targeted by the miRNAs: PTEN regulates hsa-miR-21, while TP53 regulates five miRNAs (hsa-miR-143, hsa-miR-145, hsa-miR-200, hsa-miR-215 and hsa-miR-34a) which in turn target two genes, HNF4A and MET [38].

Using the same approach for the identification of transcription factors, miRNAs, and target and host genes of miRNAs in adenocarcinoma, the regulatory associations between these elements were revealed [39]. It was found that TP53 directly regulates 9 miRNAs, consisting of hsa-miR-29b-1, hsa-miR-192, hsa-miR-194, hsa-miR-200a, hsa-miR-34a, has-miR-34b, hsa-miR-34c, hsa-miR-145 and hsa-miR-143. TP53 may also indirectly regulate additional 4 genes, CTNNB1, MET, FHIT and KRAS, by regulating these 9 miRNAs. The interaction between transcription factors and miRNAs, miRNAs and target genes and miRNAs and their host gene were investigated in human pancreatic cancer [40]. The authors described a self-adaptation association formed between TP53 and hsa-miR-125b. Also they demonstrated significant changes in expression of further 16 miRNAs in pancreatic cancer. Self-adaptation association was also described for hsa-miR-196a-1 and its host gene, HOXB7.

Beyond any doubt, such comprehensive regulatory networks associated with various types of cancer can be used in the future to develop treatments for cancer and metastasis.

## DNA methylation

Changes in methylation of the regulatory part of genes including miRNAs can serve as one of the mechanisms to change miRNA expression. It has been shown that hypermethylation of the miR-132 promoter reduces its expression, which is associated with a poor prognosis for colorectal cancer [41]. The methylated status of miR-34b and miR-34c genes is also typical of chronic lymphocytic leukemia which allows for the classification of these miRNAs as tumor suppressors [42]. It was established that methylation of the promoter of the miR-210 gene can suppress expression of this miRNA which is an important step in the process of angiogenesis [43]. Methylation of the promoters of miR-124-2, miR-218-1, miR-218-2 and miR-34b/c was markedly higher in cervical carcinoma cells with an HPV16 infection [44]. Similarly, promoter methylation of miR-33b is elevated in stomach cancer [45].

Altered methylation of miRNA encoding genes, associated with deregulated mature miRNA expression, may be related to dietary and lifestyle factors and may contribute to cancer development. In a case–control study, Cordero and colleagues [46] analyzed DNA methylation levels of miRNA encoding genes in prediagnostic peripheral white blood cells of patients with colorectal cancer or breast cancer. The researchers revealed several differentially methylated miRNA genes associated with age, sex, smoking habits and physical activity. They concluded that methylation of miRNAs detectable in the blood may represent a biomarker for the early detection or the risk of cancer. DNA methylation of miRNA promoters, such as Hsa-mir-200b cluster, may be considered as a biomarker of breast cancer subtype with prognostic value [47]. Promoter hypermethylation was shown to be one of the major mechanisms for silencing miR-31 in the triple-negative breast cancer cell lines [48]. Moreover, both miR-31 and its host gene LOC554202 are down-regulated in the TNBC cell lines of basal subtype and over-expressed in the luminal counterparts. These findings are very important for our understanding of the nature of TNBC. DNA methylation and miRNAs regulation play important role in the hereditary breast cancer [49].

Given the complex regulation of miRNA expression, the relative contributions of methylation of the promoters of miRNA genes versus their host genes, among different mechanisms is hard to evaluate. Thus, further studies on the role of methylation in the regulation of miRNA expression together with other mechanisms are necessary.

## Effects of endogenous factors and hypoxia

Various physiological and pathological stimuli, such as steroid hormones or stress, can affect miRNA expression. Several reports claimed that estrogens can affect miRNA expression [8, 50]. MiRNAs can also play an important role in the resistance of breast cancer to hormonal therapy. The empirical data of 53 research papers has been analyzed [51]. 7 miRNAs were identified (miR-10a, miR-26, miR-30c, miR-126a, miR-210, miR-342, miR-519a), the expressions of which changed after tamoxifen treatment of patients with breast cancer. Using an in silico approach, the authors have determined candidate miRNAs and their target genes that may be involved in resistance to tamoxifen. Experiments utilizing microchip technologies and RT-PCR have showed significant changes in miRNA expression in various breast cancer cell lines incubated with estradiol [52]. These findings can be important for understanding the mechanism of breast cancer’s resistance to hormonal therapy. Moreover, the spectrum of miRNA expression appears to be markedly different between ER-positive and ER-negative breast cancers [53]. Estrogen-induced miRNAs are important for adrenomodulin balance – the key regulator of cardiac activity among women [54]. MiRNA expression can also be affected by corticosterone. A study of the corticosterone-mediated depression in rats showed changes in the expression of 26 miRNAs, which can regulate closely related genes involved in the development, inflammation and depression [55]. Notably, most of the discovered miRNAs possess binding sites on the glucocorticoid receptor (GR), which suggests a common regulatory mechanism with the genes controlled by GR. The authors believe that this is a novel mechanism of GR action on the expression of miRNAs, which can aid in understanding of the development of depression. Interferon (IFN) can induce miRNA expression [56] and this serves as an important component of the host immune response during the development of malignant tumors or viral infections. For example, in many tumors the expression of miR-21 is elevated, which in most cases has oncogenic properties. Bioinformatic analysis of the miR-21 promoter has showed that STAT3 and NFκB pathways may be involved in the regulation of its expression. Yang and colleagues have also shown that upon treatment of various cell lines with IFN, the expression of miR-21 was increased three- to five fold. The authors concluded that STAT3 and the p65 subunit of NFκB can bind to the miR-21 promoter and alter its expression. Notably, differentiation of embryonic stem cells induced by retinoids is dependent on p53-regulated expression of miR-34a and miR-145, in response to DNA damaging agents [57]. While many endogenous compounds can affect miRNA expression, the precise mechanism remains to be elucidated.

Different types of hypoxia may affect microRNA expression. For example, Kiernan et al. 2016 discussed a possible role of dysregulation of microRNA expression in microglia or other CNS cells during chronic intermittent hypoxia [58]. MicroRNA expression profile also changes in tumor under the hypoxic condition. The role of miRNA in tumor microenvironment has only recently become a focus of research. Many factors such as hypoxia, variety of cell types like cancer-associated fibroblasts and macrophages may affect microRNA expression [59]. For example, in patients with breast cancer, tumor hypoxia is associated with reduced *DICER* expression and global miRNA downregulation in the microenvironment [60]. This results in the promotion of tumor progression. These findings led to a new understanding of the mechanisms of tumor progression. MiRNAs are now seriously considered as potential targets for therapy of metastasis.

## Effects of xenobiotics on miRNA expression

More and more evidence emerges demonstrating the role of miRNA in chemically induced carcinogenesis. Experimental data has shown that every carcinogen specifically affects the expression of a certain miRNA. Therefore miRNAs can serve as markers of toxicity of xenobiotics, which can be used for early diagnosis in cancer. Mechanisms explaining the changes in miRNA expression profiles under the influence of xenobiotics are widely discussed in current publications. Thus, it has been demonstrated that the expression of 5 miRNAs (miR-29b, miR-31, miR-101a, miR-130a and miR-199a-5p), changes upon treatment of embryonic fibroblasts with 2,3,7,8-tetrachlordibenzo-p-dioxin (“Agent Orange”) or under the influence of ionizing radiation [61]. Changes in the miRNA expression were observed (including 5 up-regulated and 5 down-regulated miRNAs) in hematopoietic progenitor cells in C57BL/6 mice exposed to a prolonged 4-week treatment with benzene [62], using sequencing techniques. These authors emphasize the importance of studying miRNA in hematotoxicity caused by exposure to benzene. Expression of miRNAs was also markedly affected in human hepatocytes cultured with benzo(a)pyrene. With the help of two RNA libraries, the entire transcriptome of these cells was sequenced and a novel mechanism of action of this carcinogen was proposed [63]. Aflatoxin B1, similar to benzo(a)pyrene, is a genotoxic carcinogen that can affect the profile of miRNA expression. Lu and colleagues [64], using high-throughput sequencing, have shown an increase in rno-miR-34a-5p, rno-miR-200b03p,and rno-miR-429, as well as a decrease in miR-130a-3p, in F344 rat livers treated with Aflatoxin B1. These results were confirmed by other researchers who have also shown changes in miRNA expression (especially miR-410 and miR-122) in HepaRG human hepatoma cells in response to Aflatoxin B1 and benzo(a)pyrene [65]. These data suggest that changes in the miRNA spectrum of expression can reflect the early events of non-genotoxic mechanisms of action of these genotoxic carcinogens. Expression of miRNAs under the influence of carcinogenic compounds can also change in response to diet. For example, a combination of cod liver oil and pectin, which is digested in GI tract with the subsequent release of butyrate, causes an upregulation of certain oncogenic miRNAs, whereas a diet comprised of corn oil and cellulose does not cause this effect [66]. In this study the authors have also observed changes in the expression of 26 miRNAs in intestinal stem cells. This reveals the importance of diet in the development of toxicity from certain carcinogenic compounds. The importance of the diet is confirmed by studies of dietary polyphenols with anti-oxidant activity found in many fruits, vegetables, and plants. The polyphenols can also affect the microRNA expression resulting in the up- or down-regulation of expression of genes involved in many physiological and pathological processes including cancer. Polyphenols can modulate the expression of more than 100 miRNAs important for various cellular processes such as inflammation or apoptosis [67]. The authors of the above publications noted the importance of further investigations for validation of miRNAs and their targets.

In vivo and in vitro experiments have showed that a wide range of environmental carcinogens can affect the expression of miRNAs [68]. The reversibility of an effect of chemical compounds on miRNA expression depends on the dose and duration of the exposure. Izzotti and Pulliero [69] have summarized the published studies investigating various mechanisms of carcinogenesis such as DNA damage, interaction of p53 with miRNA, mutations in miRNA genes caused by electrophilic metabolites of carcinogens and defects in DICER activity. Using in silico modeling, the authors demonstrated that mutagens can impair the process of miRNA formation from pre-miRNA. This led to the proposition that inhibition of the miRNA synthesis can be an adaptive mechanism in response to stress caused by chemical compounds.

Data on the changes in expression profiles of miRNAs in response to genotoxic (benzo(a)pyrene, Aflatoxin B1, N-nitroso-compounds, urethane) and non-genotoxic (arsenic, phenobarbital) carcinogens have been summarized [6]. In an attempt to understand the mechanism the authors propose a direct effect of carcinogens (interaction with miRNAs with the formation of adducts) and an indirect effect, for example, through inactivating the enzymatic complex DICER. The authors emphasized the importance of research, investigating the action of chemical carcinogens on miRNAs, as early markers of the carcinogenic process. Our research findings have also demonstrated that benzo(a)pyrene and DDT can affect the expression of miRNAs in the reproductive organs of female rats [70]. The majority of chemical compounds that humans and animals are exposed to on a daily basis is transformed in the liver and can therefore have a carcinogenic effect on this organ. Thus, miRNAs can play an important role in the underlying mechanism of chemically induced hepatocarcinogenesis [71]. Together this data suggests that miRNA expression can be affected by a diverse range of chemical compounds and that these effects depend on the cell type, developmental stage and gender. There is emerging evidence that exogenous factors can influence epigenetic modifications, such as DNA methylation, histone modifications and mitochondrial damage [69, 72, 73]. Gained knowledge on effects of xenobiotics on miRNA expression can also be used to assess toxic effects under chronic and acute conditions [74]. Thus, miRNAs are prospective targets in the evaluation of toxic effects of chemicals from the environment that often serve as a trigger for the development of various diseases, including cancer.

The role of miRNAs in other human pathologies is also being actively investigated with the use of animal models. It has been shown that doxorubicin causes an increase in miR-208a expression and apoptosis of cardiomyocytes in female Balb/C mice, suggesting the potential use of this miRNA for the prevention of cardiotoxicity caused by this anticancer drug [75]. New miRNAs from miR-33 family have also been identified as important regulators of cholesterol metabolism, expression of which changes upon treatment of Hep2G cells with statins [76].

Harmful habits like smoking and alcohol consumption can affect the expression of microRNAs. Alcohol dependence causes dysregulation of miR expression in human brain. It was suggested that miRNAs may have a pivotal role in the reorganization of synaptic connections and long-term neuroadaptations in alcohol dependence [77]. The recent findings suggest that chronic alcohol consumption may affect the microRNA regulating the synaptic mRNA expression [78]. Dysregulation of microRNA expression in response to alcohol was also shown in the medial prefrontal cortex of rats [79]. Elevated level of extracellular vesicles/exosomes and exosome-associated miRNA was recently found in both the serum of alcohol-fed mice and in the plasma of alcoholic hepatitis patients [80]. Alcohol induces dysregulation of miR-30a and miR-934, which may play crucial roles in the pathogenesis and progression of head and neck squamous cell carcinoma [81]. All these recent data are prompting researchers to reconsider the toxic effects of alcohol. Future investigations of the alcohol-mediated pathways in miR will help to improve our understanding of alcohol-associated diseases and advise new treatment options.

Tobacco smoking remains an important risk factor for cancer, especially for lung cancer. It was shown that cigarette smoking can change microRNA profile in many human organs and induces the change of plasma miRNA expression profiles in healthy subjects. Using microarray, Shi and co-authors [82] identified 24 up-regulated and 11 down-regulated plasma miRNAs in young healthy smokers. The authors concluded that the changes in miRNA expression profile caused by smoking can be viewed as risk factors for cancers (especially leukemia and lymphoma), cardiovascular disease, and metabolic syndrome in young adult smokers. Earlier it was also shown that serum miRNA profiles differed in nonsmokers, smokers, and lung-cancer patients [83]. The authors of this report found that let-7i-3p and miR-154-5p were significantly down-regulated in the sera of smokers and lung-cancer patients, and these changes can be considered as potential biomarkers of smoking-related lung cancer. Xi and colleagues [84] demonstrated epigenetic mechanisms that regulate miRNA expression during human pulmonary carcinogenesis and suggested utilization of chromatin remodeling agents for lung cancer therapy to restore the expression of miR-487b. The latter is up-regulated upon exposure to cigarette smoking condensate. In the last decade, a number of published articles demonstrated a strong correlation between various miRNAs, their targets and smoking-regulated genes in cancer. However, the clear understanding of the mechanisms of miR regulation by cigarette smoking is lacking so far. One of the suggested mechanisms might be the activation of Aryl hydrocarbon receptor (AhR) by tobacco smoke resulting in the changes of its target genes including ‘host’ miR genes. This is supported by the recent data of Nguyen and co-authors [85] which showed that AhR mediates the immune regulatory mechanism of rheumatoid arthritis. The authors argue that tobacco smoke is a well-known environmental risk factor for development of rheumatoid arthritis since it contains AhR agonists, such as 2,3,7,8-tetrachlorodibenzo-p-dioxin, 3-methyl cholanthrene, and benzo[a]pyrene.

Mechanisms of action of xenobiotics on miR expression can be mediated by nuclear receptors. For example, some miRs are expressed through AhR-dependent mechanism [86, 87]. It has been shown that phenobarbital-mediated activation of constitutive androstane receptors (CAR) is accompanied by the decrease in miR-122 in liver [88]. Time and dose-dependent effects of phenobarbital on the miR expression in rat liver were demonstrated recently [89]. It was also shown that expression of microRNA can be regulated by farnesoid X receptor (FXR) which is important for lipid metabolism [90]. However, future experiments will be needed to elucidate the functional relationship between nuclear receptors and miR expression.

## Conclusion

The expression of microRNAs can be regulated on multiple levels (Fig. 1). At the transcriptional level, expression of microRNA genes can change together with (intragenic miRNAs), or independently of (intergenic miRNAs), their host genes. Intergenic miRNAs have their own promoters, are expressed independently and can be regulated by separate transcription factors. In both cases, the expression of microRNA can change due to different mutations or can be regulated by methylation of the promoter. On the post-transcriptional level the expression of microRNAs can be downregulated due to changes in the activity of key miRNA biogenesis enzymes, such as Dicer and Drosha. Activity of these enzymes can also be affected by mutations or epigenetic modifications. Moreover, chemical compounds of endogenous origin (hormones, cytokines), and exogenous origin (xenobiotics), can alter microRNA expression. Activation of nuclear receptors by xenobiotics as their ligands can induce expression of both intergenic and intragenic microRNAs. All the data regarding changes in miRNA expression analyzed in this article provides insights into comprehensive mechanisms of regulation of microRNA expression, dependent on cell type, physiological conditions and external factors. It is clear that microRNAs offer potential targets for the diagnosis, prognosis and treatment of a wide variety of diseases. However in order to achieve this goal, further theoretical and experimental studies of the mechanisms of miRNA expression are crucial.
